# Association between Dietary Intake of One-Carbon Metabolism Nutrients in the Year before Pregnancy and Birth Anthropometry

**DOI:** 10.3390/nu12030838

**Published:** 2020-03-20

**Authors:** Marion Lecorguillé, Sandrine Lioret, Blandine de Lauzon-Guillain, Erwan de Gavelle, Anne Forhan, François Mariotti, Marie-Aline Charles, Barbara Heude

**Affiliations:** 1 Université de Paris, CRESS, INSERM, INRAE, F-75004 Paris, France; sandrine.lioret@inserm.fr (S.L.); blandine.delauzon@inserm.fr (B.d.L.-G.); anne.forhan@inserm.fr (A.F.); marie-aline.charles@inserm.fr (M.-A.C.); barbara.heude@inserm.fr (B.H.); 2 UMR PNCA, AgroParisTech, INRAE, Université Paris-Saclay, F-75005 Paris, France; erwan.degavelle@agroparistech.fr (E.d.G.); mariotti@agroparistech.fr (F.M.); 3 Ined, Inserm, EFS, Joint Unit Elfe, 93322 Aubervilliers, France

**Keywords:** dietary patterns, methyl-donors, one-carbon metabolism, nutrients, reduced rank regression, maternal diet, fetal growth, pre-conception period

## Abstract

Few studies have evaluated the role of methylation-pathway nutrients involved in fetal growth (B vitamins, choline, betaine, and methionine). These one-carbon metabolism (OCM) nutrients are essential for DNA methylation in the periconception period. We aimed to characterize dietary patterns of 1638 women from the EDEN mother-child cohort in the year before pregnancy according to the contribution of OCM nutrients and to study the association of such patterns with anthropometric measurements at birth. Dietary intake before pregnancy was assessed by using a semi-quantitative food frequency questionnaire. We used the reduced-rank regression (RRR) method to identify dietary patterns using OCM nutrients as intermediate variables. We ran linear regressions models to study the association between dietary patterns scores and birth weight, length, head circumference, gestational age, and sex-specific z-scores, adjusting for maternal characteristics and vitamin supplementation before and during pregnancy. Three patterns, “varied and balanced”, “vegetarian tendency”, and “bread and starchy food” were identified, explaining 58% of the variability in OCM nutrient intake. Higher scores on the “varied and balanced” pattern tended to be associated with higher birth length and weight. In mainly well-nourished young French women, we did not find evidence that variability in OCM nutrient intake has major effects on fetal growth.

## 1. Introduction 

Among the multiple factors involved in the physiological process guaranteeing a healthy pregnancy, adequate micronutrient intake during the pregnancy is necessary to support placenta homeostasis and fetal development [1,2]. Micronutrients implicated in one-carbon metabolism (OCM) deserve special attention because the OCM cycle supports multiple physiological processes essential for human development [3]. Deficiencies in micronutrients related to OCM may affect cellular metabolism, cell proliferation, and fetal growth [2]. OCM nutrients are carriers or methyl-group donors (e.g., folates, choline, betaine, methionine) or cofactors of enzymes involved in the transfer reactions of these groups to DNA (namely vitamins B2, B6, and B12) [3]. Among them, folate is a key micronutrient required for cell, placenta and brain development [4]. Choline is implicated in the synthesis of phospholipids in cell membranes and brain development [5,6]. Methionine is an essential amino acid that can be regenerated from homocysteine or choline pathways. Methionine is used to serve as the precursor to S-adenosylmethionine, the universal cellular methyl donor, and is required for protein synthesis during pregnancy [7]. Intake of these nutrients may affect epigenetic programming by changing DNA methylation in fetal tissues and placenta; therefore, they have a global impact on fetal growth [3,7]. 

Some studies have reported associations between individual OCM micronutrients and fetal development. Folate intake during pregnancy (dietary folate and folic acid supplementation) has been associated with higher birth weight. The average effect has been estimated to be a 2% increase in birth weight for every two-fold increase in folate intake [8] and further studies suggested that an adequate intake could be targeted for preventing small-for-gestational age birth weight (SGA) [9] and risk of prematurity [10]. A meta-analysis found that a low concentration of vitamin B12 (< 148 pmol/L) in maternal serum or plasma samples during pregnancy was associated with an increased risk of preterm birth (adjusted RR = 1.21 (95% confidence interval CI: 0.99, 1.49)). An increased risk of low birth weight (LBW) was also reported in association with vitamin B12 deficiency (RR = 1.15 (95% CI: 1.01, 1.31)) [11]. To date, a few studies have evaluated the role of intake of other OCM nutrients in fetal growth, with contrasting results [7,12,13,14,15,16,17]. One large study reported that higher maternal serum choline concentrations were associated with higher body fat mass (effect size = 0.60 SD (95% CI, 0.04, 1.16) per 5 μmol/L choline) and BMI z-score at birth (0.31 SD per 5 μmol/L (0.10–0.51)) [12]. An inverse association between maternal plasma betaine level and birth weight (−57.6 g per 5 μmol/L; 95% CI: −109.9, −5.3 g) was found in another study [13]. Inversely, it has been shown that choline measured in umbilical cord blood was associated with lower birth weight (−60 g per 1 SD increase in z-score; 95% CI (−89, −31) [14]. For methionine, higher concentrations measured in amniotic fluid during pregnancy was positively associated with birth weight (Spearman *r* = 0.13) but also with a lower risk of large-for-gestational age birth weight (LGA) (OR = 0.44 for 1 SD increase in plasma concentration (95 % CI: 0·21, 0·89) [16,17].

The above studies often evaluated the impact of a single nutrient on fetal growth, but recent randomized and observational studies highlighted the positive effect of multiple-micronutrient supplementation on placental vascular function and for preventing fetal growth restriction in low- and middle-income countries [2,18,19].

The OCM cycle comprises different interlinked metabolic pathways and is of interest to investigate the effect of the combination of micronutrients provided by dietary intake on fetal development [20]. The variability in food intake in a population can be summarized by dietary patterns, which have been suggested to better reflect the interactions between foods and nutrients within the total diet [21]. There are different methods to derive dietary patterns: diet quality scores or indices based on a priori knowledge of dietary guidelines, or data-driven approaches (e.g., principal component analysis) [22]. The reduced-rank regression (RRR) method combines both approaches and has been shown to provide an understanding of the pathways by which diet can influence outcomes of interest [23,24] with the use of intermediate variables (e.g., OCM nutrients) [25]. 

So far, studies investigating the role of OCM nutrients in relation to fetal development have focused on dietary intake during the pregnancy period. However, the pre-conception period also deserves attention because it is an important time window for epigenetic programming of development and health due to major epigenetics processes taking place during this period [26]. 

Therefore, after identifying dietary patterns characterizing OCM micronutrient intake (B vitamins, methionine, choline, betaine) in the year before pregnancy, our objective was first to study maternal characteristics associated with these patterns in the pre-conception period, and second to investigate their association with birth anthropometry. 

## 2. Material and Methods 

This study is reported according to the Strengthening the Reporting of Observational Studies in Nutritional Epidemiology (STROBE-nut) guidelines [27] (see Supplementary Method S1: STROBE-nut statement).

### 2.1. Study

The EDEN mother-child study is a prospective cohort that aimed to evaluate the early, pre-, and post-natal determinants of child health and development [28] (see Supporting information, data availability). Study participation was proposed to all women visiting the prenatal clinic before 24 weeks’ gestation. Between 2003 and 2006, 2002 (53%) pregnant women 18 to 45 years old were recruited in two centers: Nancy and Poitiers hospitals. Exclusion criteria were multiple pregnancies, known diabetes before pregnancy, French illiteracy or planning to move out of the region within the next 3 years. 

All subjects gave their informed consent for inclusion before they participed in the study. The study received approval from the ethics committee (CCPPRB) of Kremlin Bicêtre Hospital (02-70) and the Commission Nationale de l’Informatique et des Libertés (CNIL), the French data privacy institution. 

### 2.2. Dietary Data 

The usual diet of women during the year before pregnancy was assessed retrospectively at inclusion (before 28 weeks’ gestation; 15 weeks, on average) by using a validated food-frequency questionnaire (FFQ) [29]. This FFQ comprised 137 items, including 9 non-alcoholic and 7 alcoholic beverages; frequencies of consumption per food item was assessed on a 7-item scale from “never” to “several times a day”. Portion sizes were estimated by using pictures derived from the validated SUVIMAX (SUpplémentation en VItamines et Minéraux Anti-oXydants) portion booklet for 12 food categories (meat, French fries, pastas, vegetables, cakes, cheese etc.) on a three-level scale [30]. Daily intake (in grams/day) of each item was calculated by multiplying reported frequencies by portion sizes. Individual nutrient and total energy intake were calculated by multiplying the intake of each food item by the declared portion size and their corresponding nutrient content obtained from composition databases described below. These various intakes were estimated when less than four items were missing in the FFQ, in which case, food frequencies were imputed by the median of the observed frequencies for each item.

No food composition database was available to estimate direct intake of the nutrients implicated in OCM based on the EDEN FFQ items. Therefore, we used different sources for the food composition database and established correspondence with the Individual and National Food Consumption Survey (INCA 2) [31] to use food consumption data (see Method S2). The nutrient contents of INCA2 foods were extracted from the 2016 CIQUAL food composition database [32] that we used for estimating intake of energy, macronutrients, and B vitamins (B2, B6, B9, B12). To assess methionine intake, we used existing estimates for the INCA2 food items that were provided by a recent study and derived from French published analyses and international databases [33]. Finally, we used a specific food composition table from the US Department of Agriculture (USDA) National Nutrient Database for assessing choline and betaine intake [34], following a methodology similar to that used for methionine (see Method S3). 

When we matched the EDEN FFQ food items and the INCA2 food items, we sometimes had to combine several INCA2 items to characterize a more generic EDEN FFQ one (i.e., various types of beef). In these cases, we used information for the frequency of food consumption by adult French women 18 to 50 years old for each of these food items in the INCA2 study [31] to compute an average weighted nutrient composition for the corresponding EDEN one.

These weights were then applied in estimating each nutrient or vitamin intake per EDEN FFQ item (see Method S2).

### 2.3. Other Collected Data

We collected parity, gestational age, child sex, and anthropometry at birth from medical records. We calculated *z*-scores for birth weight, length, and head circumference according to the French Audipog reference [35], which accounts for gestational age at birth and sex. Infants with a weight z-score strictly lower than the 10th percentile or strictly higher than the 90th percentile were classified as small for gestational age (SGA) or large for gestational age (LGA), respectively. Premature birth was defined as gestational age < 37 weeks’ gestation. 

Sociodemographic and pregnancy characteristics were recorded by interviews with the mothers by a midwife investigator at 6 months of pregnancy and after delivery. Maternal characteristics studied were country of birth (Europe, outside Europe), maternal age (18–25, 25–29, 30–34, ≥ 35 years), employment status (employed/student/staying at home, parental leave, unemployed, or other), maternal education level (at most lower secondary, upper secondary, post-secondary, or tertiary (Bachelor’s, Master’s, or doctoral level) according to the International Standard Classification of Education [36]), living with a partner (yes vs. no), and monthly household income (<1501, 1501–2300, 2301–3000, > 3000 €/month). Health-related variables included smoking during pregnancy and were classified into three groups to identify non-smokers, moderate, and heavy smokers (never, 1–9 cigarettes/day, ≥ 10 cigarettes/day). Pre-pregnancy body mass index (BMI) was calculated as reported weight (kg) divided by measured height^2^ (m^2^) and classified into 4 categories according to the World Health Organization thresholds: underweight (<18.5 kg/m^2^), normal weight (18.5 to <25 kg/m^2^), overweight (25.0 to <30 kg/m^2^), and obese (≥ 30 kg/m^2^). 

Information on dietary supplements was collected from maternal self-completed questionnaires: at inclusion for supplementation in the 3 months before pregnancy and after delivery for supplementation during pregnancy. We had information on consumption of mixed multivitamins/minerals or the use of single micronutrients, among which we selected vitamins B2, B6, and B9 (folic acid).

We classified the variable “vitamin supplementation” into the following four categories: no supplementation, supplementation only before pregnancy, supplementation only during pregnancy, and supplementation before and during pregnancy. 

### 2.4. Sample Selection

**Figure 1:**
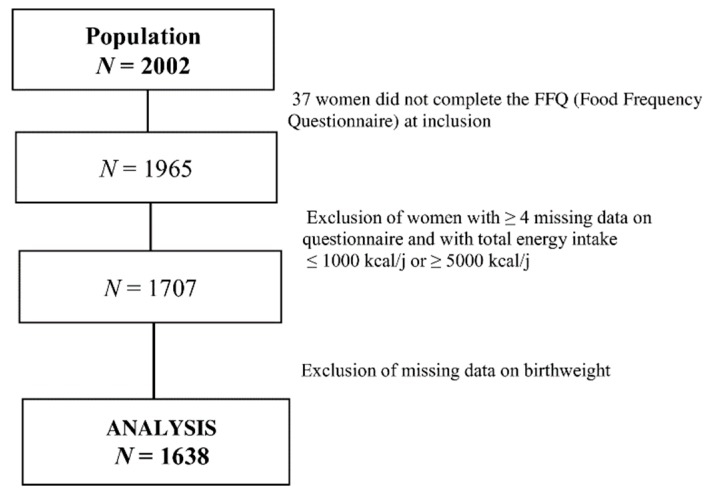
Flow chart of the selection of women included in the analysis.

Mothers who did not complete the FFQ (*n* = 37), with more than 3 items missing (*N* = 155), with implausible food group values (*N* = 2), or with extreme total energy intake ≤ 1000 or ≥ 5000 kcal/day (*N* = 101) were excluded from the RRR analysis. Those for whom infant birth weight was not available (early termination of pregnancy, delivery outside the study hospitals or lost to follow-up) were excluded (*N* = 69) from subsequent analyses. Figure 1 summarizes the steps of the population selection; 1638 women were included in the final multivariate analysis.

### 2.5. Missing Data Imputation 

We performed multiple imputation of missing data for confounders and outcomes (birth length and head circumference) (Table S1). We included all variables providing information for the imputation process (sociodemographic factors, medical information, and birth characteristics) after ranking them in ascending order of missing data. We assumed that data were missing at random and generated 5 independent imputed datasets by using the fully conditional specification method. The results of the imputed datasets were combined by using the SAS “MI analyze” procedure, and standard errors were calculated by using Rubin’s rules, which take into account the variability between the multiple regressions in imputed datasets [37,38]. Categorical variables were imputed by using a multinomial model, binary and ordinal variables by logistic regression, and continuous variables by linear regression.

### 2.6. Statistical Analysis 

#### 2.6.1. Dietary Patterns 

The RRR method was used to determine the linear combinations of predictors (food groups) that explain as much as possible of the variation in the response, or nutrients variables (also called “intermediate” variables). Before using the RRR method, the 137 food items in the EDEN cohort were grouped into 36 major food groups or specific food items for those that were particularly rich in specific OCM nutrients, such as whole grain bread, avocado, and broccoli (Table S2). More than half of the sample consumed at least one food of the 36 food groups.

We used the 36 food items as predictor variables and the log-transformed nutrient variables (folates; vitamins B12, B2, and B6; betaine; choline; and methionine) as response variables. Confounding by total energy intake is a major point to address because women who consume more energy may also have higher consumption of specific nutrients [39]. Therefore, we pre-adjusted nutrient intake (response variables) for total energy intake and used the residuals of the regression models for each nutrient in the RRR analysis. The analysis was conducted using the proc pls and “RRR” option in SAS [23]. Factor loadings above a threshold of 0.20 were used to characterize the seven dietary patterns derived. For each woman, a score was calculated for each dietary pattern; it was the sum of the z-standardized average daily intake of the 36 food items multiplied by the corresponding weight provided by the RRR analysis. 

#### 2.6.2. Descriptive Statistics

Characteristics of mothers and their children are described with mean ± SD and n (%) before imputation. Unadjusted associations between demographic, socioeconomic and health maternal characteristics, and the scores for each selected dietary pattern were tested by ANOVA, with *p* < 0.05 considered statistically significant.

#### 2.6.3. Association with Outcomes

We analyzed the association between selected dietary pattern scores and birth weight, length, and head circumference z-scores (hereafter called “birth weight, length or head circumference at birth”) and gestational age by multivariable linear regression. Because the effect of micronutrient intake might differ whether or not they were provided by supplementation, interaction terms between the pattern scores and maternal supplement intake before pregnancy were systematically tested (in the complete-cases analysis). We also tested interactions terms between each pattern scores and offspring sex (in the complete cases analysis). No interaction terms were significant neither for birth weight nor for birth length (*p* > 0.05).

We also assessed the association between vitamin supplementation and infant anthropometry at birth. Logistic regression was used for analyzing the risk of SGA (as compared with non-SGA) or LGA (as compared with non-LGA), and prematurity. We adjusted gestational age and prematurity for infant sex. Betas (β) or odds ratios (ORs) and 95% confidence intervals (CIs) were calculated per 1 SD of the dietary pattern score. 

Potential confounding factors were selected from the literature and their bivariate associations with our factors of interest. Therefore, all models were adjusted for center, maternal education level, maternal age, employment status, monthly household income, parity, smoking during pregnancy, BMI, and vitamin supplementation before and during pregnancy if appropriate. Maternal BMI was taken into account with a power three factor. As a complementary analysis, we used dietary pattern score quintiles to illustrate the shape of the association with birth size. 

Finally, as a sensitivity analysis, we re-ran all the analyses restricted to complete cases (i.e., without any missing data for exposure, outcome, and covariates).

## 3. Results 

### 3.1. Population Characteristics 

The characteristics of the women selected for analysis (*N* = 1638) are summarized in Table 1. Mean maternal age was 30 years and women were mainly born in Europe (97%) and were employed (77%). Many had a tertiary education level (34%). About 26% of mothers smoked during pregnancy and more than 26% were overweight or obese at the beginning of pregnancy. Only 8.7% of women received vitamin and/or mineral supplementation within the three months before pregnancy and about 26% received supplementation only during pregnancy. 

### 3.2. Dietary Pattern Scores

Three of the seven patterns resulting from the reduced rank regression explained 58% of the considered variation in intake of nutrient (response) variables (Table 2). The first pattern, “varied and balanced”, was characterized by a high intake of low-fat milk, meat, liver, fish, eggs and egg dishes, cereals, mixed vegetables, chicory, leeks and cabbage, and broccoli and a low intake of snacks and confectionery and sugar-sweetened beverages. This pattern was characterized by high positive coefficients for B vitamins, choline, and methionine (0.37–0.44), but a lower coefficient for betaine (0.16) (Table 3). The second pattern, “vegetarian tendency”, explaining 14.5% of the variation in intake of nutrient variables, was characterized by a high intake of fruits, chicory, mixed vegetables, cereals, and bread, and a low intake of meat and liver. This pattern had a positive and high response score for B6 and B9 vitamins and betaine (0.38–0.51) and negative coefficients for vitamin B12, choline and methionine. The third pattern, “bread and starchy food”, explaining 8.3% of response variation, was characterized by a high intake of bread, sandwiches and rice, pasta and other grains, and a low intake of low-fat milk, fruits, fruit juices, and sugar-sweetened beverages. This pattern had a high coefficient for betaine (0.84) and negative coefficients for vitamins B2, B6, and B9. The other profiles are not presented because they did not display a specific pattern and each explained less than 4% of the variation in intake of nutrient variables. As a complementary result, Table S3 described the OCM nutrient intake (mean ± SD) per tertile of each dietary pattern, and shows as reference the current French and international (for choline) recommendations.

### 3.3. Associations between Maternal Characteristics and Dietary Patterns 

Younger women and those with a lower education level or who were unemployed had lower scores for the “varied and balanced” pattern than their counterparts, whereas women with a high monthly household income had higher scores for this pattern (Table 4). We found consistent associations for the “vegetarian tendency” pattern, except for maternal age, which was not associated with this pattern. In addition, students and primiparous women more frequently adhered to this pattern. Women with low maternal education or aged <25 years old had low scores on the “bread and starchy food” pattern. Smoking was associated with low scores on the “vegetarian tendency” and “varied and balanced” patterns. Underweight women less frequently ate a varied and balanced diet, whereas overweight women had low scores on the “vegetarian tendency” pattern. Women with vitamin supplementation before pregnancy had high scores on the “varied and balanced” pattern and had relatively high scores on the “vegetarian tendency” pattern compared to non-supplemented women (*p* = 0.05).

### 3.4. Association of Dietary Pattern Scores in the Year before Pregnancy with Birth Anthropometric Outcomes

**Figure 2:**
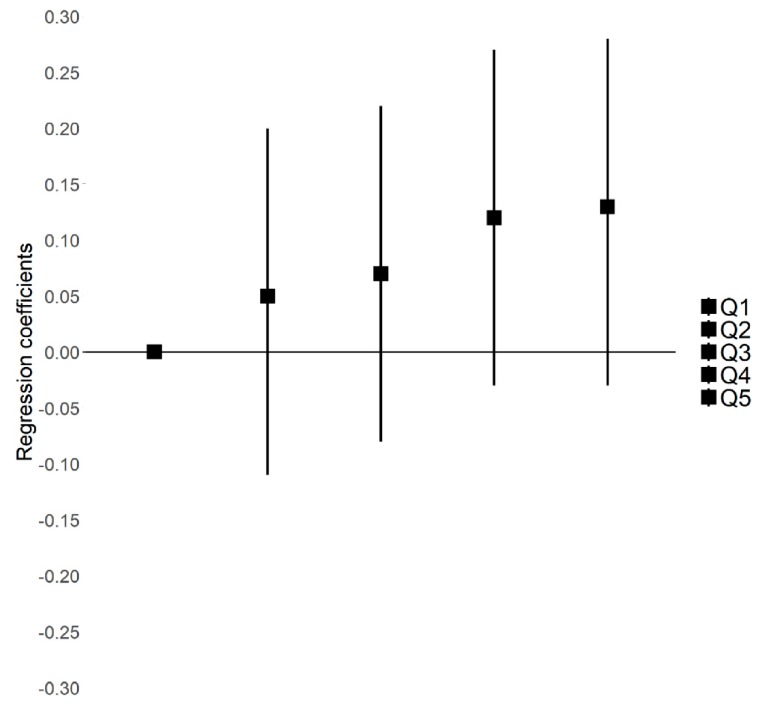
Linear regressions for birth length z-score by quintiles of “varied and balanced” pattern scores: analysis after multiple imputation (*N* = 1638). Q1 to Q5 are quintiles 1 to 5. Q1 (first quintile) is the reference. *p*-trend = 0.06. Model adjusted for center, maternal education level, maternal age, employment status, monthly household income, parity, smoking during pregnancy, body mass index, and vitamin supplementation. Birth length z-score according to the Audipog reference [35] β [95% CI].

On unadjusted linear regression, adherence to the “varied and balanced” pattern was positively associated with both birth weight (*p* = 0.02) and birth length (*p* < 0.01), and positively but not significantly associated with head circumference at birth (*p* = 0.06) (Table 5). After adjustment for maternal characteristics and pregnancy-related factors, the association with the “varied and balanced” diet decreased for birth weight (*p* = 0.14), birth length (*p* = 0.08), and head circumference at birth (*p* = 0.43) (Table 5). Using quintiles of scores for dietary patterns (Figure 2), we found that birth length increased across quintiles of the “varied and balanced” pattern (*p* for trend = 0.06).

On unadjusted logistic regression, the “bread and starchy food” pattern was associated with reduced probability of SGA (*p* = 0.05) and high scores on the “varied and balanced” pattern were associated with increased risk of LGA (*p* < 0.01). These associations were still significant in the adjusted model (Table S4). We found no significant association between the “vegetarian tendency” pattern and birth anthropometry. In the adjusted analyses, high scores on the “vegetarian tendency” pattern were associated with a low risk of prematurity (*p* = 0.03). 

In adjusted analyses, vitamin supplementation during the three months before pregnancy was not significantly associated with birth size. Gestational length was shorter on average for women with supplementation before pregnancy (*p* = 0.05), but the risk of prematurity was not significantly higher (Table S4). We found no significant interactions between vitamin supplementation before pregnancy and dietary pattern scores in association with birth anthropometry (all *p* > 0.05).

Consistent results were found in the complete-case analysis, although with weaker associations between the “varied and balanced” pattern and both birth weight (*β* = 0.02 [95% CI −0.02;0.06]) and birth length (*β* = 0.03 [95% CI −0.01; 0.07]). 

## 4. Discussion

This study is the first attempt to evaluate the extent to which dietary patterns of OCM nutrient intake in the year before pregnancy are associated with fetal growth for women of childbearing age. The dietary patterns derived with the RRR method, with vitamins B2, B6, B9, and B12; choline; betaine; and methionine as intermediate variables, explained a substantial part (more than 58%) of the variation in intake of these micronutrients before pregnancy. Importantly, our study identified three patterns of maternal diet as the main source of OCM variability. A high score on the “varied and balanced diet” pattern correspond to a high intake of the main OCM micronutrients and a likely adequation with recommended intake for major OCM nutrients. The “vegetarian-tendency” pattern is related to high intake of vitamin B6, B9, and betaine to the detriment of others nutrients such as vitamin B12, choline, and methionine. The “bread and starchy food” pattern, is mainly characterized by betaine intake and a low level of B9 vitamins.

The nutrient intake according to dietary patterns that we found is consistent with previous studies showing that a high intake of plant-based foods may lead to high folate levels [40]. The INCA2 study showed that participants belonging to “traditional” or “diversified” dietary clusters had a greater vitamin intake, whereas adults with processed food or sandwich consumption had a low vitamin intake [41]. Our patterns were consistent with the reported dietary sources of choline (eggs, meat, wheat germ) and betaine (wheat, bread, spinach) [42]. 

The association of dietary patterns with socioeconomic and other behaviors was consistent with results from other studies of dietary patterns of young pregnant or non-pregnant women [43,44,45]. Consumption of fruits, vegetables, whole grains, and fish was high in women with increased education [43,45]. Age was also positively associated with a dietary pattern characterized by high consumption of fruits and vegetables [45]. Smoking and a high BMI were negatively related to fruit intake [43]. 

Importantly, our study further focused on dietary patterns that explained the variability in intake of the OCM nutrients combined and fetal development during the pre-conception period, which is a crucial time window for fetal programming. Some studies showed the association of single OCM micronutrients provided by the maternal diet or supplementation with both brain development and optimal birth weight [5,8,11,46]. However, single OCM nutrient studies do not take into account the associations between one OCM nutrient and the other OCM nutrients, although they collectively play a role in the underlying metabolism. 

Our results suggest little evidence for an association between the “varied and balanced diet” score before pregnancy and birth weight, length, and head circumference. A high adherence to this pattern was also associated with an increased risk of LGA, which might be related more to longer length than increased adiposity. However, we found no relation between scores on the “vegetarian tendency” pattern and anthropometric measurements at birth. A pre-pregnancy varied and balanced diet providing a variety of nutrients may be best to support fetal development. Conversely, the vegetarian tendency pattern, although characterized by high intake of vitamins B6 and B9 also featured low vitamin B12 consumption, which may be less optimal for fetal growth. These results are consistent with studies highlighting the effect of multiple micronutrient supplementation for fetal growth [18,19]. 

Our finding of a high adherence to the “vegetarian tendency” pattern associated with reduced risk of prematurity is consistent with a review showing the importance of adequate folate status to avoid risk of prematurity [10]. However, another meta-analysis did not find any beneficial effects of folate supplementation during pregnancy on length of gestation [8]. 

The third pattern, “bread and starchy food,” contributed mainly to the variability in betaine intake and was associated with reduced risk of SGA not modified by adjustments for confounders. This pattern was less associated with maternal characteristics. Conversely, two studies showed that plasma betaine concentration during the second trimester was associated with smaller infant birth size, birth weight, and less abdominal fat mass [13,16]. Few other studies have evaluated the role of betaine for fetal growth, and controversial results might be explained by differences in measures of betaine intake or chance findings.

Although our dietary patterns were driven by the variability in content of OCM nutrients in the maternal diet, other aspects of the diet (macronutrients, fibers…) may also explain the observed associations, especially for the “varied and balanced” and “vegetarian tendency” patterns. 

We did not find any association between vitamin supplementation and anthropometry at birth. Only a few women (8%) had vitamin supplementation before pregnancy and maintained this consumption during pregnancy. We did not have information on the duration of supplementation and whether it concerned specific trimesters during pregnancy. This lack of information precluded a more detailed analysis of the different vitamins or minerals used for supplementation. However, we found a positive association between vitamin supplementation before pregnancy and high scores on the “varied and balanced” pattern. A study of the NutriNet-Santé cohort showed that dietary supplement users had a high dietary intake of most vitamins and minerals [47]. 

Pre-conceptional vitamin supplementation was significantly associated with reduced gestational length, three days on average, with no association with risk of prematurity. This finding may be explained by reverse causation: women more at risk of gestational complications are more likely to be followed before pregnancy and then initiate early vitamin supplementation. 

### 4.1. Strengths and Limitations

This study provides new insights into the characteristics of the diet of women of childbearing age before pregnancy in light of its contribution to the combined intake of micronutrients essential for fetal development. As compared with other dietary pattern approaches, the RRR technique was particularly useful because of its multivariate approach combined with existing knowledge of diet–disease associations and may help provide a novel understanding of the pathways by which diet influences health. Associations between food groups and nutrients can be used to interpret the effects of food groups as components of dietary patterns. We also used a multiple imputation technique to limit bias due to missing data for our variables of interest and confounders.

Among the limitations is the questionable accuracy of self-reported data on diet before pregnancy and supplementation before pregnancy and during pregnancy. Women may have under- or over-reported their consumption of specific food groups (e.g., social desirability for the diet or dietary habits). However, the accuracy might be similar for information collected by a health professional. 

In our study, women with disadvantaged social status and possibly more nutritional deficiencies were under-represented, which may explain in part why we did not find stronger associations with fetal growth. Moreover, residual confounding cannot be ruled out, and thus, these results need replication.

### 4.2. Perspectives

The role of nutrition in OCM and DNA methylation has been more extensively studied in animal models than epidemiological studies. However, recent human research has indicated that altered consumption of methyl donors may affect the fetal epigenome [7]. One study conducted in Gambia found that infants conceived during the dry or wet season (with significant seasonal variation in nutrients) had different levels of DNA methylation at a number of sites correlated with their methyl-donor micronutrient status [26,48]. In another study, maternal diet and supplemental intake of methyl-group donors in the periconception period were associated with modifications in DNA methylation of buccal cells in genes selected for their involvement in growth and metabolism [49]. New evidence has demonstrated that epigenetic mechanisms may be involved in early development but also in later health and disease development [50]. Therefore, the associations between dietary patterns and placental and cord blood methylation need further study. The replication of results for the association between OCM nutrients on fetal growth and the study of more specific aspects of development, such as neural development, are also necessary. 

## 5. Conclusions

This study identified three patterns describing the variability of the combined intake of OCM nutrients in the diet of French women of childbearing age. High adherence to a pattern associated with the combined intake of all major OCM nutrients may have a small contribution to fetal linear growth. In this sample of French women without large nutritional deficiencies, we did not find any evidence that the variation in intake of micronutrients implicated in methylation-related pathways, have major effect on fetal growth.

## Figures and Tables

**Table 1:** Characteristics of women selected for analysis (*N* = 1638).

Sociodemographic Data	Missing Data n (%)	Population
Recruitment center, n (%)	0 (0)	-
Poitiers		809 (49.4)
Nancy		829 (50.6)
Maternal age (years), mean ± SD	0 (0)	29.7 ± 4.9
Country of birth, n (%)	108 (6.6)	-
Europe		1489 (97.3)
Maternal education level, n (%)	12 (0.7)	-
Lower secondary school		421 (25.9)
Upper secondary school		291 (17.9)
Post-secondary		367 (22.6)
Tertiary		547 (33.6)
Employment status, n (%)	13 (0.8)	-
Employed		1245 (76.6)
Student		43 (2.6)
Staying at home		337 (20.7)
Monthly household income (euros), n (%)	9 (0.5)	-
<1501		246 (15.1)
1501–2300		463 (28.4)
2301–3000		438 (26.9)
> 3000		482 (29.6)
Living with a partner (Yes), n (%)	9 (0.5)	880 (54.0)
Smoking before pregnancy (Yes), n (%)	20 (1.2)	-
No		1040 (64.3)
1–9/day		209 (12.9)
At least 10/day		369 (22.8)
Pregnancy		
Gestational age (Weeks) *, mean ± SD	0 (0)	39.2 ± 1.7
Primiparous (Yes), n (%)	2 (0.12)	731 (44.7)
Vitamin supplementation, n (%) No Before pregnancy During pregnancy Before and during pregnancy	12 (0.73)	- 930 (57.2) 142 (8.7) 423 (26) 131 (8.1)
Smoking during pregnancy (Yes), n (%)	41 (2.5)	-
No		1185 (74.2)
1–9/day		338 (21.2)
At least 10/day		74 (4.6)
Pre-pregnancy BMI, n (%)	33 (2.0)	-
Underweight (<18.5 kg/m^2^)		134 (8.3)
Normal (18.5 to <25 kg/m^2^)		1060 (66)
Overweight (25.0 to <30 kg/m^2^)		289 (18)
Obesity (≥30 kg/m^2^)		122 (7.6)
Child sex, n (%)	0 (0)	-
Male		867 (52.9)
Female		771 (47.1)
Birth weight z-score ^1^, mean ± SD	0 (0)	−0.02 ± 1.0
Birth length z-score ^1^, mean ± SD	47 (2.9)	−0.01 ± 1.0
Head circumference z-score ^1^, mean ± SD	97 (5.8)	0.04 ± 0.8
Small for gestational age ^2^, n (%)	0(0)	157 (9.6) -
Large for gestational age ^2^, n (%)	0(0)	121 (7.4)
Prematurity (<37 Weeks) *, n (%)	0(0)	91 (5.6)

* WA, weeks’ gestation. ^1^ Birth weight z-score according to the French Audipog reference [35]. ^2^ Birth weight categories to assess fetal growth: small for gestational age (<10th percentile) or large for gestational age (>90th percentile), according to the French Audipog reference [35].

**Table 2:** Reduced rank regression factor loadings for the dietary patterns in the year before pregnancy in the EDEN cohort (*N* = 1707): selection of the first three dietary patterns.

Food Groups	Dietary Pattern 1	Dietary Pattern 2	Dietary Pattern 3
Explained Variation ª	35	14	8
Low-fat milk	0.31 *	−0.04	−0.26 *
Other vegetables	0.31 *	0.33 *	0.00
Fish	0.29 *	−0.06	0.17
Meat	0.29 *	−0.32 *	0.20
Chicory	0.26 *	0.28 *	0.03
Leek, cabbage	0.23 *	0.16	−0.01
Eggs and egg dishes	0.23 *	−0,13	0.05
Cereals	0.22 *	0.36 *	−0.07
Broccoli	0.21 *	0.15	0.01
Liver	0.21 *	−0.28 *	−0.01
Fruits	0.19	0.41 *	−0.27 *
Water	0.16	0.11	0.02
Soya	0.16	0.11	−0.03
Whole grain bread	0.10	0.18	0.16
Hight-fat milk and cream	0.08	0.04	−0.24
Coffee, tea	0.07	0.00	−0.05
Avocado	0.06	0.11	−0.04
Bread	−0.006	0.21 *	0.53 *
Boiled or baked potatoes	0.04	0.00	0.00
Cheese	0.03	−0.12	−0.10
Rice, pasta, and others	0.03	0.06	0.36 *
Sauces	0.01	0.05	0.02
Nuts and seeds	−0.003	0.12	−0.12
Cold meats	−0.004	−0.18	0.02
Fruit juice	−0.02	0.18	−0.31 *
Ready-to-eat foods	−0.02	−0.09	0.13
Honey, jam	−0.04	0.09	0.04
Ice cream	−0.05	−0.01	−0.09
Fried or roasted potatoes	−0.08	−0.14	−0.03
Chocolate bar	−0.09	−0.05	−0.11
Butter	−0.10	−0.00	0.04
Alcohol	−0.10	−0.03	−0.03
Sandwich	−0.11	−0.04	0.22 *
Cakes, pastry	−0.19	−0.01	0.04
Snacks and confectionery	−0.20 *	−0.003	−0.10
Sugar-sweetened beverages	−0.29 *	−0.09	−0.23 *

ª Explained Variation in all responses or nutrients variables from the reduced-rank regression, * Factor loading ≥ 0.20.

**Table 3:** One-carbon metabolism nutrient intake in the year before pregnancy and association with the three dietary patterns resulting from the reduced-rank regression analysis in the EDEN cohort (*N* = 1707).

		Associations with Dietary Patterns **		
	Pre-conception Nutrient Intake *	Varied and Balanced	Vegetarian Tendency	Bread and Starchy Food
Vitamin B2 (mg/day)	2.27 ± 0.9	0.41	0.01	−0.33
Vitamin B6 (mg/day)	1.92 ± 0.8	0.44	0.38	−0.13
Vitamin B9 (µg/day)	379.47 ± 165	0.37	0.51	−0.30
Vitamin B12 (µg/day)	6.06 ± 3.7	0.37	−0.46	0.09
Choline (mg/day)	386.89 ± 149	0.43	−0.22	0.11
Betaine (mg/day)	198.42 ± 77.3	0.16	0.42	0.84
Methionine (mg/day)	2.09 ± 0.9	0.39	−0.40	0.23

* mean ± SD value of nutrient consumption during the year before pregnancy. ** coefficients of the responses (nutrients variables) scores for the three dietary patterns. A high response score for the “varied and balanced” factor reflects a diet rich in major OCM nutrients excepted for betaine.

**Table 4:** Unadjusted associations between maternal characteristics and dietary patterns (*N* = 1638).

Maternal Characteristics		Varied and Balanced β (95% CI)	Vegetarian Tendency β (95% CI)	Bread and Starchy Food β (95% CI)
**Maternal age**	<25	−0.34 [−0.51; −0.17]	−0.03 [−0.19; 0.13]	−0.16 [−0.30; −0.02]
	25–29	0 (reference)	0 (reference)	0 (reference)
	30–34	0.10 [−0.03; 0.23]	0.01 [−0.11; 0.13]	−0.08 [−0.19; 0.03]
	35	0.17 [0; 0.33]	0.04 [−0.11; 0.19]	−0.01 [−0.14; 0.13]
**Country of birth**	Non-Europe	0.27 [−0.09; 0.63]	−0.05 [−0.47; 0.38]	0.18 [−0.09; 0.46]
	Europe	0 (reference)	0 (reference)	0 (reference)
**Maternal education level**	Lower secondary school	−0.29 [−0.43; −0.14]	−0.42 [−0.55; −0.29]	−0.29 [−0.41; −0.17]
	Upper secondary school	−0.15 [−0.31; 0.01]	−0.25 [−0.39; −0.10]	−0.16 [−0.29; −0.02]
	Post-secondary	−0.13 [−0.28; 0.02]	−0.16 [−0.30; −0.03]	0 [−0.12; 0.12]
	Tertiary	0 (reference)	0 (reference)	0 (reference)
**Employment status**	Yes	0 (reference)	0 (reference)	0 (reference)
	Student	−0.04 [−0.38; 0.31]	0.38 [0.07; 0.69]	−0.17 [−0.46; 0.11]
	Staying at home	−0.22 [−0.36; −0.08]	−0.19 [−0.32; −0.07]	−0.11 [−0.22; 0.01]
**Monthly household income (euros)**	<1501	−0.08 [−0.25; 0.10]	−0.31 [−0.47; −0.15]	−0.1 [−0.25; 0.05]
	1501–2300	−0.04 [−0.19; 0.11]	−0.17 [−0.31; −0.04]	−0.2 [−0.32; −0.08]
	2301–3000	0 (reference)	0 (reference)	0 (reference)
	>3000	0.18 [0.04; 0.33]	0.07 [−0.07; 0.20]	−0.03 [−0.15; 0.1]
**Smoking during pregnancy**	No	0 (reference)	0 (reference)	0 (reference)
	1–9/day	−0.21 [−0.34; −0.07]	−0.2 [−0.32; −0.07]	−0.03 [−0.14; 0.08]
	At least 10/day	−0.65 [−0.92; −0.39]	−0.64 [−0.88; −0.40]	−0.11 [−0.34; 0.11]
**Living with a partner**	No	−0.11 [−0.22; 0]	−0.03 [−0.13; 0.07]	−0.04 [−0.13; 0.05]
	Yes	0 (reference)	0 (reference)	0 (reference)
**Parity**	Multiparous	0.01 [−0.10; 0.12]	−0.26 [−0.36; −0.16]	−0.01 [−0.10; 0.09]
	Primiparous	0 (reference)	0 (reference)	0 (reference)
Pre-pregnancy BMI *	Underweight	−0.44 [−0.65; −0.24]	−0.03 [−0.21; 0.16]	0.01 [−0.16; 0.18]
	Normal	0 (reference)	0 (reference)	0 (reference)
	Overweight	−0.01 [−0.16; 0.13]	−0.16 [−0.30; −0.03]	−0.1 [−0.22; 0.02]
	Obesity	−0.01 [−0.22; 0.20]	−0.34 [−0.54; −0.15]	−0.03 [−0.20; 0.15]
**Vitamin supplementation**	No	0 (reference)	0 (reference)	0 (reference)
	Before pregnancy	0.31 [0.11; 0.51]	0.18 [0; 0.36]	0.03 [−0.14; 0.20]
	During pregnancy	0.1 [−0.04; 0.23]	0.11 [−0.01; 0.23]	0.06 [−0.05; 0.17]
	Before and during pregnancy	0.11 [−0.10; 0.32]	0.13 [−0.06; 0.32]	−0.02 [−0.19; 0.15]

95% CI, 95% confidence interval. * Pre-pregnancy BMI: underweight as defined <18.5 kg/m^2^; normal weight 18.5 to <25 kg/m^2^; overweight 25.0 to <30 kg/m^2^; obese ≥30 kg/m^2^.

**Table 5:** Association between dietary patterns in the year before pregnancy or vitamin supplementation and neonatal anthropometry at birth or gestational age (*N* = 1638) after multiple imputation: unadjusted and adjusted models.

Dietary Patterns	Birth Weight z-score β (95% CI) *		Birth Length z-score β (95% CI) *		Head Circumference z-score β (95% CI) *		Gestational Age (Weeks) β (95% CI)
	Unadjusted	Adjusted ^a^	Unadjusted	Adjusted ^a^	Unadjusted	Adjusted ^a^	Unadjusted ** Adjusted **^,a^
**Varied and balanced**	**0.05 [0.01; 0.09]**	0.03 [−0.01; 0.07]	**0.07 [0.03; 0.11]**	0.04 [−0.01; 0.08]	0.03 [−0.002; 0.07]	0.01 [−0.02; 0.05]	−0.06 [−0.13; 0.02] −0.05 [−0.12; 0.02]
**Vegetarian tendency**	0.01 [−0.03; 0.06]	0.01 [−0.04; 0.05]	0.03 [−0.02; 0.08]	0.01 [−0.04; 0.06]	0.02 [−0.02; 0.06]	0.01 [−0.03; 0.05]	0.05 [−0.03; 0.13] 0.04 [−0.04; 0.12]
**Bread and starchy food**	0.02 [−0.03; 0.07]	0.02 [−0.03; 0.07]	−0.03 [−0.08; 0.02]	−0.02 [−0.07; 0.03]	−0.03 [−0.07; 0.02]	−0.03 [−0.08; 0.01]	0.06 [−0.03; 0.15] 0.05 [−0.04; 0.14]
**Vitamin** **supplementation**							
No	0 (reference)	0 (reference)	0 (reference)	0 (reference)	0 (reference)	0 (reference)	0 (reference) 0 (reference)
Before pregnancy	−0.09 [−0.26; 0.08]	−0.09 [−0.26; 0.08]	−0.15 [−0.34; 0.03]	−0.12 [−0.30; 0.05]	−0.08 [−0.23; 0.07]	−0.08 [−0.22; 0.07]	−0.30 [−0.60; −0.004] −0.33 [−0.63; −0.02]
During pregnancy	−0.06 [−0.17; 0.05]	−0.08 [−0.19; 0.03]	−0.07 [−0.19; 0.05]	−0.07 [−0.19; 0.04]	−0.04 [−0.14; 0.06]	−0.07 [−0.17; 0.03]	−0.08 [−0.27; 0.12] −0.11 [−0.30; 0.09]
Before and during pregnancy	0.04 [−0.14; 0.22]	0.07 [−0.11; 0.24]	0.01 [−0.19; 0.19]	0.03 [−0.15; 0.22]	−0.02 [−0.18; 0.13]	−0.04 [−0.20; 0.12]	−0.04 [−0.35; 0.27] −0.05 [−0.36; 0.27]

^a^ adjusted for center, maternal education level, maternal age, employment status, monthly household income, parity, smoking during pregnancy, body mass index, and vitamin supplementation. * Birth z-scores according to the French Audipog reference [35]. ** Gestational age: adjusted for infant sex. β values of linear regression and 95% CIs for 1 SD of dietary pattern score.

## Supplementary Materials

The following are available online at https://www.mdpi.com/2072-6643/12/3/838/s1, Table S1: Details of the method used for multiple imputation (*N* = 1638), Table S2: Composition of the 36 food groups considered for generating dietary patterns with the reduced-rank regression method, Table S3: Mean ± SD of OCM nutrients in the year before pregnancy according to tertiles of each pattern and comparison with recommendations (when they are known), Table S4: Association between dietary patterns in the year before pregnancy or vitamin supplementation and risk of small-for-gestational age (SGA) or large-for-gestational age (LGA) and prematurity (*N* = 1638) after multiple imputation: unadjusted and adjusted models, Method S1: STROBE-nut statement, Method S2: French assignment between INCA2 food item and EDEN food-frequency questionnaire (FFQ) and calculation of the OCM nutrients intake, Method S3: Choline and betaine intake assessment.

## Abbreviations

BMI, body mass index; CI, confidence intervals; DP, dietary pattern; FFQ, food-frequency questionnaire; OCM, one-carbon metabolism; LBW, low birth weight; LGA, large-for-gestational age; OR, odds ratios; RRR, reduced rank regression, SGA small-for-gestational age; USDA, US Department of Agriculture.
