# Supplementary material for: Association between Dietary Intake of One-Carbon Metabolism Nutrients in the Year before Pregnancy and Birth Anthropometry
Source: Nutrients. 2020 Mar 20;12(3):838. doi: 10.3390/nu12030838 (PMC7146458; doi:10.3390/nu12030838)
Supplement: Supplementary file 1 [file nutrients-12-00838-s001.zip › Supplementary files/Method S1.docx]

Method S1: French assignment between INCA2 food item and EDEN food-frequency questionnaire (FFQ) and calculation of the OCM nutrients intake

The EDEN FFQ comprises 137 food items containing single foods, food groups (same variety) or main recipes. The French INCA2 survey was performed in 2006-2007 with a nationally representative sample of French people, and food consumption was recorded by 7-day dietary records and comprised 1342 items.

To assess maternal nutrient intake, including vitamins, from the EDEN FFQ, we derived a specific composition database by using consumption data based on the French INCA2 food consumption survey [31]. Because the INCA2 table contains more food items than the EDEN FFQ (1342 vs 137), we followed the two steps described below:

**Assignment of INCA2 food items to the 137 EDEN food items**

1) We assigned the INCA2 food item to the corresponding EDEN one when they were similar.

2) When several INCA2 items corresponded to the same generic EDEN food item, we assigned all the related foods from the INCA2 study to the EDEN item (e.g., various types of lamb meat in INCA2 assigned to a generic lamb meat item in EDEN).

We used 885 INCA2 foods for this correspondence.

**Calculation of the nutrient contents of the 137 EDEN food**

When several INCA2 items were assigned to a single EDEN FFQ item, we computed a weighted mean nutrient and energy composition. Weights were based on the average intake frequency of each of the corresponding INCA2 item by French women 18 to 50 years old, in the 7-day food records of the INCA2 survey [31].

We calculated the weights as follows:

$W_{j}=\frac{C_{j}}{\sum_{k=1}^{n_{i}} C_{k}}$ (1)

where i is the EDEN FFQ item, ni the total number of INCA2 items assigned and j one of these INCA2 items, cj is the average frequency intake of the j item estimated from the INCA2 survey.

The weighted means for energy and nutrients were calculated as follows

$N_{i}=\sum_{j=1}^{ni} (W_{j}*N_{j})$ (2)

Where Nj is the nutrient composition of the jth item.

Nutrient content was assessed as the following units:

B2 into mg/100 g of food

B9 into ug/100g of food

B12 into ug/100g of food

B6 into mg/100 g of food

Methionine into mg/100 g of food
