# Supplementary material for: Association between Dietary Intake of One-Carbon Metabolism Nutrients in the Year before Pregnancy and Birth Anthropometry
Source: Nutrients. 2020 Mar 20;12(3):838. doi: 10.3390/nu12030838 (PMC7146458; doi:10.3390/nu12030838)
Supplement: Supplementary file 1 [file nutrients-12-00838-s001.zip › Supplementary files/Table S1.docx]

**Table S1**: Details of the method used for multiple imputation (N=1638)

| Variable | Type of variable | Model used to predict data* | Missing data % |
| --- | --- | --- | --- |
| Recruitment center  Child’s sex  Parity  Universal health coverage  Complementary health coverage  Living with a partner  Vitamins supplementation  Country of birth | Binary  Binary  Binary  Binary  Binary  Binary  Binary  Binary | Logistic regression  Logistic regression  Logistic regression  Logistic regression  Logistic regression  Logistic regression  Logistic regression  Logistic regression | 0%  0%  0.12%  0.5%  0.55%  0.55%  0.6%  6.6% |
| Dietary Pattern 1 (scores)  Dietary Pattern 2 (scores)  Dietary Pattern 3 (scores)  Dietary Pattern 4 (scores)  Dietary Pattern 5 (scores)  Dietary Pattern 6 (scores)  Dietary Pattern 7 (scores)  Birth weight  Maternal age  Gestational age at birth  Maternal weight before pregnancy | Continuous  Continuous  Continuous  Continuous  Continuous  Continuous  Continuous  Continuous  Continuous  Continuous  Continuous | Linear regression  Linear regression  Linear regression  Linear regression  Linear regression  Linear regression  Linear regression  Linear regression  Linear regression  Linear regression  Linear regression | 0%  0%  0%  0%  0%  0%  0%  0%  0%  0%  0.8% |
| Maternal height  Body mass index  Gestational weight gain  Maternal weight at 20 years  Birth length  Head circumference at birth  Placental weight  Gestational diabetes  Gestational hypertension  Monthly household income  Maternal education  Employment situation  Smoking before pregnancy  Smoking during pregnancy | Continuous  Continuous  Continuous  Continuous  Continuous  Continuous  Continuous  Categorical (3 categories)  Categorical (3 categories)  Categorical (4 categories)  Categorical (4 categories)  Categorical (3 categories)  Ordinal (3 categories)  Ordinal (3 categories) | Linear regression  Linear regression  Linear regression  Linear regression  Linear regression  Linear regression  Linear regression  Multinomial regression  Multinomial regression  Multinomial regression  Multinomial regression  Multinomial regression  Logistic regression  Logistic regression | 1.3%  2.0%  2.0%  3.5%  2.9%  5.8%  26%  0.1%  0.1%  0.55%  0.7%  0.8%  1.2%  2.5% |

*Fully conditional specification method.
