# Supplementary material for: Association between Dietary Intake of One-Carbon Metabolism Nutrients in the Year before Pregnancy and Birth Anthropometry
Source: Nutrients. 2020 Mar 20;12(3):838. doi: 10.3390/nu12030838 (PMC7146458; doi:10.3390/nu12030838)
Supplement: Supplementary file 1 [file nutrients-12-00838-s001.zip › Supplementary files/Table S2.docx]

**Table S2**: Composition of the 36 food groups considered for generating dietary patterns with the reduced rank regression method

|  | Group | Composition |
| --- | --- | --- |
| 1 | Cheese | Seven type of cheese (emmental, gouda, processed etc.) |
| 2 | Low-fat milk | Cottage cheese or yogurt 0% fat, semi-skimmed milk, skimmed milk |
| 3 | Hight-fat milk and cream | Yogurt, dessert, pudding, cream fluid heavy or light |
| 4 | Rice, pasta and other grains | Rice, pasta, wheat germ |
| 5 | Bread | Bread, crackers |
| 6 | Whole grain bread | Whole grain bread |
| 7 | Cereals | Breakfast cereals high-fibre or not, cereal bar fruits |
| 8 | Cakes, pastry | Pastry, pancakes, cake, tarts |
| 9 | Sandwich | Sandwich |
| 10 | Fruits | Kiwi, apricot, peaches, strawberries, bananas, lemon, orange, peer, apple, grape, other fruits |
| 11 | Chicory | Chicory, spinach, lettuce, endive |
| 12 | Leek, cabbage | Leek, cabbage, cauliflower |
| 13 | Broccoli | Broccoli |
| 14 | Avocado | Avocado |
| 15 | Other vegetables | Carrots, beans, peas, peppers, tomato, corn, other vegetables |
| 16 | Soya | Soya, tofu |
| 17 | Boiled or baked potatoes | Potatoes baked, dauphine, mashed |
| 18 | Fried or roast potatoes | Fried or roast potatoes |
| 19 | Eggs and egg dishes | Eggs and egg dishes |
| 20 | Meat | Beef, pork, veal, lamb |
| 21 | Liver | Liver, tongue, kidney |
| 22 | Cold Meats | Dry sausage, salami, ham, terrine |
| 23 | Fish | Fish smoked, breaded, salted, shellfish |
| 24 | Ready-to-eat | Tart, pizza, hamburger, hot-dog, chili con carne, fish meals, ravioli |
| 25 | Butter | Butter, margarine |
| 26 | Ice-cream | Ice cream |
| 27 | Sauces | Ketchup, vinegar, mayonnaise |
| 28 | Fruit juice | Apple juice, fruit cocktail |
| 29 | Sugar-sweetened beverages | Coca cola, soda, limonade |
| 30 | Water | Water |
| 31 | Alcohol | Alcoholic beverage: Beer, cider, wine, gin, rum |
| 32 | Coffee, tea | Coffee, tea |
| 33 | Snacks and confectionery | Chips, biscuits, cookies, crackers, candies |
| 34 | Chocolate bar | Chocolate bar, cocoa pure powder |
| 35 | Honey, jam | Honey, jam, chocolate spread with hazelnuts |
| 36 | Nuts and seeds | Nuts, peanuts, dried fruits |
