# Supplementary material for: Association between Dietary Intake of One-Carbon Metabolism Nutrients in the Year before Pregnancy and Birth Anthropometry
Source: Nutrients. 2020 Mar 20;12(3):838. doi: 10.3390/nu12030838 (PMC7146458; doi:10.3390/nu12030838)
Supplement: Supplementary file 1 [file nutrients-12-00838-s001.zip › Supplementary files/Table S3.docx]

**Table S3**. Mean ± sd of OCM nutrients in the year before pregnancy according to tertiles of each patterns and comparison with recommendations (when they are known)

| **N=1707** |  | Mean of nutrients ± sd | | |
| --- | --- | --- | --- | --- |
|  |  | **Varied and balanced** | | |
|  | **Recommandations** | 1^st^ tertile | 2^nd^ tertile | 3^rd^ tertile |
| **Vitamin B2** (mg/day) | 1.5 mg/day^1^ | 1.9 ± 0.7 | 2.1 ± 0.7 | 2.8 ± 0.9 |
| **Vitamin B6** (mg/day) | 1.5 mg/day^1^ | 1.6 ± 0.6 | 1.7 ± 0.6 | 2.4 ± 0.8 |
| **Vitamin B9** (µg/day) | 400 μg/day^1^ | 319.6 ± 133.9 | 345.9 ± 125.0 | 472.9 ± 186.8 |
| **Vitamin B12 (**µg/day) | 4 ug/day^1^ | 4.8 ± 2.3 | 5.5 ± 2.3 | 7.9 ± 5.0 |
| **Choline** (mg/day) | 425 mg/day^2^ | 328.8 ± 118.0 | 359.6 ± 111.1 | 472.3 ± 169.9 |
| **Methionine** (mg/day) | - | 1.8 ± 0.74 | 1.9 ± 0.7 | 2.5 ± 1.0 |
| **Betaine** (mg/day) | - | 188.1 ± 76.2 | 186.2 ± 63.1 | 221.0 ± 85.8 |
|  |  |  |  |  |
|  |  |  | **Vegetarian tendency** |  |
|  |  | 1^st^ tertile | 2^nd^ tertile | 3^rd^ tertile |
|  |  |  |  |  |
| **Vitamin B2** (mg/day) |  | 2.3 ± 0.9 | 2.1 ± 0.8 | 2.4 ± 0.9 |
| **Vitamin B6** (mg/day) |  | 1.8 ± 0.7 | 1.7 ± 0.6 | 2.3 ± 0.8 |
| **Vitamin B9** (µg/day) |  | 323.1 ± 141.6 | 342.1 ± 125.1 | 473.1 ± 181.3 |
| **Vitamin B12 (**µg/day) |  | 7.51 ± 5.0 | 5.4 ± 2.3 | 5.3 ± 2.5 |
| **Choline** (mg/day) |  | 422.1 ± 174.0 | 353.5 ± 121.4 | 385.1 ± 137.7 |
| **Methionine** (mg/day) |  | 2.4 ± 1.01 | 1.9 ± 0.7 | 1.9 ± 0.7 |
| **Betaine** (mg/day) |  | 171.5 ± 70.4 | 187.0 ± 64.6 | 236.8 ± 80.4 |
|  |  |  |  |  |
|  |  |  | **Bread and starchy food** |  |
|  |  | 1^st^ tertile | 2^nd^ tertile | 3^rd^ tertile |
|  |  |  |  |  |
| **Vitamin B2** (mg/day) |  | 2.5 ± 0.9 | 2.2 ± 0.8 | 2.1 ± 0.8 |
| **Vitamin B6** (mg/day) |  | 2.0 ± 0.8 | 1.8 ± 0.7 | 1.9 ± 0.8 |
| **Vitamin B9** (µg/day) |  | 419.7 ± 191.2 | 365.7 ± 143.3 | 353.0 ± 149.5 |
| **Vitamin B12 (**µg/day) |  | 6.05 ± 3.9 | 5.7 ± 2.6 | 6.4 ± 4.2 |
| **Choline** (mg/day) |  | 382.6 ± 140.6 | 375.1 ± 138.8 | 402.9 ± 164.7 |
| **Betaine** (mg/day) |  | 2.0 ± 0.8 | 2.0 ± 0.8 | 2.3 ± 1.0 |
| **Methionine** (mg/day) |  | 160.0 ± 66.0 | 195.7 ± 66.9 | 239.6 ± 76.9 |
|  |  |  |  |  |

^1^Recommendations from the French Agency for Food, environmental and Occupational Health & Safety (https://www.anses.fr/fr/system/files/NUT2017SA0141.pdf): recommendations for adult population >18 years old.

^2^Recommendations from the USDA (https://www.nal.usda.gov/sites/default/files/fnic_uploads//390-422_150.pdf).
