# Supplementary material for: Association between Dietary Intake of One-Carbon Metabolism Nutrients in the Year before Pregnancy and Birth Anthropometry
Source: Nutrients. 2020 Mar 20;12(3):838. doi: 10.3390/nu12030838 (PMC7146458; doi:10.3390/nu12030838)
Supplement: Supplementary file 1 [file nutrients-12-00838-s001.zip › Supplementary files/Table S4.docx]

**Table S4**: Association between dietary patterns in the year before pregnancy or vitamin supplementation and risk of small for gestational age (SGA) or large for gestational age (LGA) and prematurity (N=1638) after multiple imputation: unadjusted and adjusted models.

|  |  | **SGA OR (95% CI) *** | |  | **LGA OR (95% CI) *** | |  | **Prematurity OR (95% CI)** |
| --- | --- | --- | --- | --- | --- | --- | --- | --- |
| **Dietary patterns** | N** | Unadjusted model | Adjusted model^a^ | N | Unadjusted model | Adjusted model^a^ | N | Unadjusted Adjusted model^b^ |
| Varied and balanced | 157 | 0.96 [0.83;1.11] | 1.00 [0.86;1.16] | 121 | 1.22 [1.05;1.41] | 1.19 [1.02;1.39] | 91 | 1.15 [0.97;1.36] 1.10 [0.92;1.32] |
| Vegetarian tendency | 157 | 1.01 [0.86;1.18] | 1.02 [0.87;1.21] | 121 | 0.96 [0.81;1.14] | 0.97 [0.81;1.16] | 91 | 0.86 [0.71;1.05]  **0.79 [0.65;0.98]** |
| Bread and starchy food | 157 | **0.84 [0.70;1.0]** | **0.83 [0.70;0.99]** | 121 | 0.98 [0.81;1.19] | 1.00 [0.82;1.23] | 91 | 1.01 [0.8;1.26] 1.02 [0.81;1.28] |
| **Vitamin supplementation** |  |  |  |  |  |  |  |  |
| No | 82 | 0 (reference) | 0 (reference) | [66-68] ** | 0 (reference) | 0 (reference) | 46 | 0 (reference) 0 (reference) |
| Before pregnancy | 16 | 1.3 [0.74;2.29] | 1.25 [0.7;2.23] | [12-13] | 1.31 [0.7;2.44] | 1.25 [0.64;2.43] | 9 | 1.29 [0.62;2.7] 1.30 [0.61;2.76] |
| During pregnancy | 50 | 1.38 [0.95;2.0] | 1.46 [0.99;2.14] | [33-35] | 1.14 [0.74;1.75] | 1.09 [0.70;1.71] | 27 | 1.29 [0.79;2.11] 1.31 [0.79;2.16] |
| Before and during pregnancy | 9 | 0.77 [0.38;1.57] | 0.74 [0.36;1.54] | 8 | 0.86 [0.4;1.83] | 0.95 [0.43;2.06] | 9 | 1.44 [0.69;3.02] 1.33 [0.62;2.85] |

^a^Adjusted for centre, maternal education level, maternal age, employment status, monthly household income, parity, smoking during pregnancy, body mass index and vitamin supplementation.

^b^Additional adjustment for infant sex

*Risk of SGA birth weight (<10^th^percentile) and risk of LGA birth weight (>90^th^percentile), according to the French Audipog reference [35].

** Minimum–maximum number of women in each category of variable depending on imputed table
