# Supplementary material for: Association between Dietary Intake of One-Carbon Metabolism Nutrients in the Year before Pregnancy and Birth Anthropometry
Source: Nutrients. 2020 Mar 20;12(3):838. doi: 10.3390/nu12030838 (PMC7146458; doi:10.3390/nu12030838)
Supplement: Supplementary file 1 [file nutrients-12-00838-s001.zip › Supplementary files/Method S2.doc]

**Method S2: Choline and betaine intake assessment**

We created a specific food composition database for choline and betaine because their contents were not available in the 2016 CIQUAL database. This was done for the 885 INCA2 food items for which we calculated a weight assigned to the 137 EDEN FFQ items.

To do so we applied the same methodology as developed by de Gavelle et al. to assess methionine intake [33]. This team has established a food correspondence between INCA2 food items and several international databases including the US Department of Agriculture Nutrient Database (USDA), the only source available for choline and betaine food content.

We selected as priority data those from the USDA database derived from analytical data. The analytical data are values from published literature or unpublished laboratory reports. They can be inserted into the USDA database because they are, or after selection, averaging, or a weighted combination of analytical values. Non-analytical data were kept as a second choice.

If we found several foods from the USDA database corresponding to a single INCA2 item, we kept all the foods assigned and used the mean value.

To assign USDA food items to the 885 INCA2 items used to calculate nutrient content for the EDEN FFQ, we followed the steps described below:

*Step 1: Correspondence between USDA and INCA2 food items*

This process ended with different level of correspondence:

**a) Direct correspondence with the USDA database: 44.3% (N=392)**

**b) Assignment of similar food items: 44.5% (N=394)**

When there was no exact correspondence between USDA and INCA2 food, we used “similar or approximate food items” from the USDA database:

- Other brand, or raw (for some fish without cooked item with choline or betaine contents)
- Similar meat from another species: i.e., beef kidney for pork kidney
- General item (bread, dried sausage, puff pastry roll, cheese, chocolate, cakes) for some typical specific French food that do not exist in the USDA database
- Different brands for some foods
- General items for alcohol beverages (specific French kind of beer, wine)

**c) Estimation for recipe: 1.8% (N=16)**

When French recipes from INCA2 study did not exist in the USDA database, we selected the main ingredients from the USDA table and computed a mean value to estimate choline and betaine

**d) No assignment (9.3%)**

- No weighting available from the INCA2 study

- Specific vegetables or soup not found in the USDA database

**e) Assignment to O for foods containing no or very little choline (N=1) and betaine (N=76):**

In case of missing data on choline and betaine composition in the USDA table for food containing no or very little amount of these two nutrients, we set the value to 0 (e.g., cocoa powder, sugar, water, sorbet, coffee, tea)

**f) Different foods for betaine intake**

For several USDA foods, we had information for choline but not betaine. Thus, the USDA food assigned may be different for choline and betaine intake (N=114) and there is a higher percentage of no assignment (19.6%).

*Step 2: Calculation of the choline and betaine contents of foods*

We used the same approach than the previous used to calculate the nutrient contents in Method S1. We calculated mean of choline and betaine intake for each of the 137 EDEN FFQ food groups. When several INCA2 food items were assigned to a single EDEN item, if betaine or choline intake was not available for all the INCA2 items, we re-estimated the weighting using only INCA2 foods with available information for choline and betaine.

Nutrients units:

Choline into mg/100 g of food

Betaine into mg/100 g of food

(https://www.ars.usda.gov/ARSUserFiles/80400525/Data/Choline/Choln02.pdf)
